# On Minimum Parking Space Required by Automatic Parallel Parking

**DOI:** 10.3390/s22030795

**Published:** 2022-01-20

**Authors:** Xuan Wang, Momiao Zhou, Zhengqiong Liu, Zhizhong Ding

**Affiliations:** School of Computer and Information, Hefei University of Technology, Hefei 230009, China; 2020111044@mail.hfut.edu.cn (X.W.); mmzhou@hfut.edu.cn (M.Z.); zqliu@hfut.edu.cn (Z.L.)

**Keywords:** automatic parallel parking, minimum parking space, intelligent vehicles

## Abstract

Automatic parking system has been widely equipped on manual driving vehicles for decades. Due to the limited environment sensing ability and not-accurate-enough positioning system, however, it usually requires a very large redundant space for a car to accomplish one successful parking maneuver. There would be two differences in the coming era of automatic driving: (1) sufficiently accurate perception and positioning systems can be expected to be available, and (2) the number of parking trials could be large when parking without passengers on the vehicle. Under such circumstances, it is quite important for the vehicle to know exactly whether the external and internal spaces are large enough for parking and how the minimum number of trials will be required. This paper presents closed-form solutions of minimum internal and external space required for automatic parallel parking and the corresponding trials if the parking is based on circular arcs. On the other hand, it is also very useful for the vehicle to know the required minimum space if only one trial is allowed, for which the solutions are presented both for circular arcs and continuous-curvature curves based parking, respectively. To the best of our knowledge, there is no so far systematic analysis and research on the issue addressed in this paper.

## 1. Introduction

In populated big cities like Beijing, Tokyo, Shanghai, etc., the rapid growth in car ownership not only increases the burden of urban traffic but also exacerbates the problem of insufficient road-side parking space. In the whole driving process, for drivers, parking is often the most difficult part, especially in the case that available parking space is relatively narrow, which is easy to cause minor collisions and scuffing [1,2]. According to statistics, 23% of car accidents are related to parking collisions [3]. In this sense, automatic parking is deemed as the most promising technology to solve these problems [4,5]. An efficient automatic parking system can free the driver from the pressure of parking operation and complete the parking maneuver quickly, while greatly reducing the probability of accidents during the process.

The existing automatic parking system is often designed for non-fully automatic fuel vehicles, which still have some issues to be tackled. Due to the imperfection of the system’s parking space detection, real-time environmental perception capabilities, and accuracy in vehicle control, it is often necessary to add some redundant space for parking space to ensure that the vehicle does not collide or scuff with the parking boundary or surrounding obstacles [6,7]. That is to say, the existing parking slots provided in cities are usually larger than the vehicle demand in theory. With the development of Intelligent Transportation System (ITS), the future fully-automatic intelligent vehicles can obtain more detailed and accurate information of parking slot and surrounding obstacle from the road-side units (RSU) [8,9], and with the assistance of more advanced radar and video detection technology, the accuracy of parking space detection and distance measurement will be effectively improved [10]. Besides, the accuracy of future fully-automatic intelligent vehicles in terms of trajectory tracking and control will be much more precise than the traditional manually driven vehicles or semi-autonomous vehicles [11]. In this context, the automatic parking system can complete parking operations safely and precisely and thus the requirement for redundant space can be greatly reduced. For example, the driver can stay outside and park the vehicle remotely. In this case, the complexity of the parking trajectory and driving comfort are not the key matters. What we really care about is the minimum space required for the parking task. Meanwhile, the parking space as a system target can be composed of parking slot-markings on the road surface or be created as a form of open spaces without the slot-markings by adjacent obstacles such as vehicles, walls, or curbs. As long as the target space is greater than the minimum space required by automatic parking, the space can be used to complete the parking task, which will improve the utilization of the open space. In addition, due to the limited road space, the space available for the vehicle to adjust its attitude before entering the parking space is very limited on narrow roads, and thus the determination and calculation of this part of space is also quite necessary. Consequently, the determination and calculation of the minimum internal and external parking space required for automatic parking (internal space refers to the size of the parking slot and external space refers to the space occupied during the parking maneuver before the vehicle enters the parking slot) is an essential prerequisite for future automatic parking systems.

### 1.1. Related Works

Since automatic parallel parking is more challenging than perpendicular parking and skewed parking in calculating the minimum parking space, and more importantly, the shortage of roadside parking resources is more serious, in order to reduce the occupation of road space, most roadside parking spaces are designed as parallel parking slots. Thus, this paper discusses the minimum internal and external space required for automatic parallel parking and gives an analytical solution with closed-form for the minimum space required for some typical parking methods. The analysis of the minimum parking space is usually based on parking methods where easy geometric equations are involved. Some researchers have constructed parking trajectories based on mathematical models of classical curves [12,13,14,15] and gave solutions for the space required for their parking methods. In [16], the minimum length of parking slot required for parking based on admissible circular arcs was investigated. The minimum slot depends on the initial position of the vehicle, which leads to a conclusion that is not generalizable. As the research progressed, [17] used the geometric method based on the reverse procedure of retrieving a vehicle from the parking slot proposed by [18] to give the minimum length of parking slot, which only depends on the characteristics of the vehicle. This conclusion is general and based on the assumption that the turning radius of the second part of the parking maneuver is the minimum turning radius, whereas the radius of the first part can be any value greater than the minimum one. But the drawback is that the width of the parking slot is regarded as equal to the width of the vehicle, which does not take into account the necessity for lateral obstacle avoidance during vehicle movement and is clearly unreasonable. Later in [19], this drawback was considered and a solution for the minimum width of the parking slot was given. It should be emphasized that these conclusions are both based on the assumption that vehicle only parks in one trial (without longitudinal velocity sign-changing) and the parking trajectories are composed of admissible circle arcs. After that, [20] considered the longitudinal and lateral safety redundancy distances of parking slot, and when the redundancy distance takes the value of 0, the minimum parking slot is consistent with the conclusions given by [19]. However, it is such a pity that all the papers mentioned above only consider the minimum parking slot (i.e., internal parking space), without taking into account the need for external parking space.

These methods based on admissible circular arcs have the disadvantage of discontinuous curvature. When faced with the curvature discontinuity point, the vehicle must stop and reorient the front wheel, which will involve undesirable time delay and damage to the steering column. Therefore, some smooth curves were introduced, which can be divided into two categories. One is parametric curves whose curvature is a polynomial function of their arc length, e.g., clothoids [15,21], cubic spirals [22], or more recent $\eta^{4}-\mathrm{spline}$ [23]. The other is the curves whose coordinates can be expressed in a closed-form, e.g., B-splines [24], quintic polynomials [25], or polar-splines [26]. The former category is used more often. Ref. [27] proposed the first continuous curvature path planner that considers the maximum curvature derivative constraint, and the curves generated by this planner are called Simple Continues Curvature (SCC) curves. In [28], the range of values for the minimum parking slot length required for parking based on SCC curves was given, but the minimum parking slot width is not discussed. Later in [29], the authors introduced a linearly steering spiral to the Reeds and Sheep (RS) curve [12] to generate a smooth path for parking and discussed the minimum length of parking slot in two cases according to whether the curvature of the path at the end of the parking is 0 or not, and the corresponding conclusion was given.

In the work of defining and calculating the minimum parking space, these aforementioned papers only intuitively consider the limitation of the length of the parking slot, without considering the width of the parking slot and the external parking space, which are also very important for automatic parallel parking systems. In addition, all their conclusions are given on the premise that the vehicle must be parked into slot in one trial. In practice, however, n-trial (n>1) and one-trial parking are both significant. If comfort or convenience is the first concern, one-trial parking is more suitable; while if space-saving is the first concern, n-trial parking is absolutely necessary [30]. Nevertheless, no researcher has systematically studied the parking space required for n-trial parking. In view of this, the study of the minimum space required by automatic parallel parking still has huge room for enhancement.

### 1.2. Contributions of This Paper

This paper fills the gap in the solution of parking space for the previous related works. The solution of parking slot width and the external parking space is often neglected by researchers. In this paper, the internal and external space required for n-trial parking and the neglected parts for typical one-trial parking methods are solved systematically. The results are presented as closed-form solutions and are only related to the characteristics and mechanical properties of the vehicle, which can be widely extended to different practical application scenarios. At last, the minimum parking space required for the various parking methods discussed in this paper is compared, and suggestions are made for selecting specific parking methods based on actual parking needs.

The contributions of this paper are as follows:

1. Considering the extreme case that the number of trials is unbounded, we derive the minimum internal and external space required for n-trial automatic parallel parking based on circular arcs with closed form. The optimal n is given, and it is shown that the lower limits of the minimum internal and external space both approximate the size of the vehicle.
2. Considering the extreme case that only one trial is allowed, we give the minimum internal and external space required for automatic parallel parking based on circular arcs and continuous-curvature curves, respectively. It is shown that although the continuous-curvature curve has more smooth transition for path than circular arc, it requires larger space for parking.
3. The minimum internal and external spaces that are derived can be the guidelines for urban planners to design right-sized parking slots, and for vehicles to estimate whether a certain piece of space is sufficient for automatic parking with its preferred parking methods.

The rest of this paper is organized as follows. In Section 2, some preliminary definitions are given and the geometric models related to the kinematics of the vehicle are presented. Section 3 gives the minimum internal and external space required for n-trial automatic parallel parking based on circular arcs. In Section 4 and Section 5, the minimum internal and external space required for one-trial parking based on circular arcs and continuous-curvature curves are given, respectively. Finally, Section 6 contains the conclusions.

## 2. Preliminary Modeling

### 2.1. Simplified Model of Vehicle and Parking Slot

The vehicles studied in this paper are small and medium-sized cars such as Compact, Hatchback, Sedan, etc., whose outer contours can be approximately modeled as a rectangle, including the outside rear-view mirrors. The vehicle is symmetric around the central axis. The maximum left steering angle is equal to the maximum right steering angle, so that the vehicle has the same left and right turning radius. The parking slot is a rectangular area with three-side barriers. The external space required for parking is also considered as a rectangular area for simplicity. The initial posture of the vehicle is assumed to be parallel to the parking slot. The whole parking maneuver should be within both the parking slot (no collision with barriers) and the external rectangular area.

**Figure 1:**
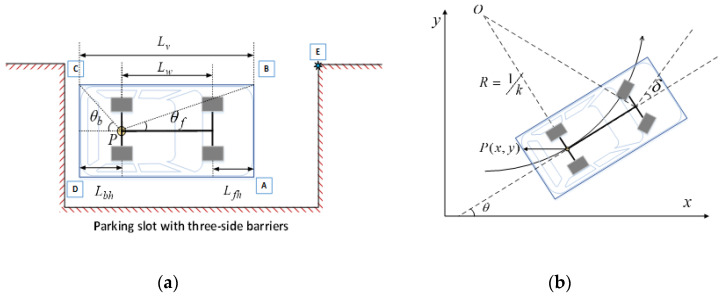
Simplified model of vehicle and parking slot (**a**); and vehicle kinematic model (**b**).

As shown in Figure 1, the four vertices of the vehicle rectangle are respectively represented by A, B, C, and D (A is the right front corner). The length and width of the vehicle rectangle is $L_{v}$ and $W_{v}$, respectively; the wheelbase is $L_{w}$ and the track is $L_{b}$; the front overhang is $L_{fh}$ and the rear is $L_{bh}$; the midpoint of the rear axle of the vehicle is denoted as P, which is referred to as the reference point.

### 2.2. Kinematic Model of Vehicle

The kinematic model of the vehicle is usually used to analyze the motion state of the vehicle during the parking maneuver. The parking process is usually performed at low speed, assuming that the vehicle is absolutely rigid body, in regardless of the effect of tire slippage. In addition, two front wheels are directional and the rear wheel’s axle is fixed with the vehicle body. The motion of the vehicle conforms to Ackermann steering geometry at any moment. Therefore, all wheels turn around the instantaneous center of the rotation (denoted as $O_{l}$/$O_{r}$ for left/right steering), which is on the line collinear with the rear axle of the vehicle. The distance from the reference point P to the instantaneous center of rotation is the instantaneous turning radius, denoted as $R_{p}$. The motion model of the vehicle can be expressed as

(1) $$\left(\begin{array}{l} \dot{x}\\ \dot{y}\\ \dot{\theta }\\ \dot{k} \end{array}\right)=\left(\begin{array}{c} \cos\theta \\ \sin\theta \\ k\\ 0 \end{array}\right)v+\left(\begin{array}{l} 0\\ 0\\ 0\\ 1 \end{array}\right)\sigma$$

where (x,y) is the position of the reference point; θ represents the orientation of the vehicle and k indicates the curvature, which is the inverse of the turning radius. ($\dot{x}$, $\dot{y}$, $\dot{\theta }$ and $\dot{k}$ are the derivatives of x, y, θ, k with respect to time t) The two inputs v and σ are the longitudinal velocity and the angular acceleration at the reference point (x,y) respectively. Denote the steering angle as δ and its derivative as $\dot{\delta }$. Then the following relationship can be obtained:

(2) $$k=\frac{\tan\delta }{L_{w}}=\frac{1}{R_{p}},\;\sigma =\dot{k}=\frac{\dot{\delta }}{L_{w}\cos^{2}\delta }\;.$$

Obviously, when the vehicle moves at a constant steering angle, then k is fixed and there is $\dot{k}=0$. The trajectory of the vehicle follows a circle arc. Consequently, the motion model can be simplified to

(3) $$\left(\begin{array}{l} \dot{x}\\ \dot{y}\\ \dot{\theta } \end{array}\right)=\left(\begin{array}{l} \cos\theta \\ \sin\theta \\ \frac{\tan\delta }{L_{w}} \end{array}\right)v$$

It can be observed from (2) that if the steering angle reaches the upper bound $\delta_{\max}$, then the turning radius of the reference point reaches the minimum, denoted as $R_{p}^{\min}$. Indeed, $R_{p}^{\min}$ is just the minimum turning radius of the vehicle in this paper, which can be expressed as

(4) $$R_{\min}=R_{p}^{\min}=\frac{L_{w}}{\tan\delta_{\max}}$$

## 3. Minimum Space for N-Trial Parking Based on Circle Arcs

**Figure 2:**
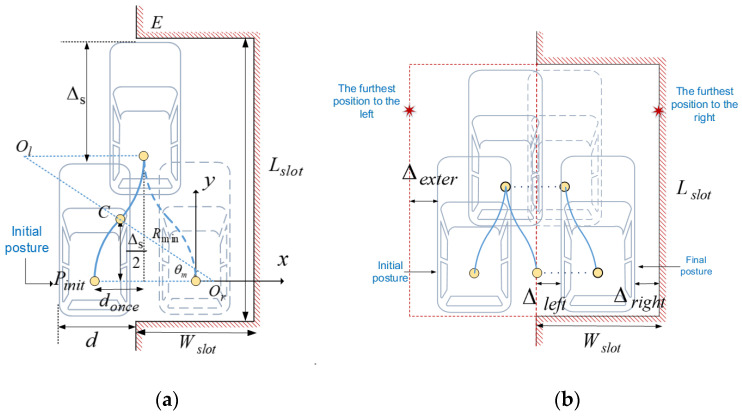
S-shaped forward and backward movement (**a**); and schematic representation of the space required for parking (**b**).

In some cases, n-trial parking is needed for space saving. In this section, we explore the theoretical minimum space required for automatic parallel parking based on circular arcs, with unbounded number of trials. Here a trial refers to an S-shaped forward or backward movement, the trajectory of which is composed by two symmetric circle arcs. First, it is assumed that the vehicle is located just alongside the bottom of the parking slot as the initial posture, as shown in Figure 2a. At the end of each movement, the vehicle is parallel to the parking slot. Each movement produces a certain lateral displacement, and eventually the vehicle can be completely parked into the slot after multiple phases of displacement. In order to maximize the lateral displacement of each movement, the steering angle is set to $\delta_{\max}$, i.e., the radius of every circle arc is $R_{\min}$. Based on these assumptions, the theoretical minimum internal and external space required for automatic parallel parking can be obtained, which is given in Theorem 1.

**Theorem** **1.** *The minimum internal and external space required for automatic parallel parking based on circular arcs can be obtained by the following formula:*

(5) $$L_{slot}^{\min}=L_{exter}^{\min}=L_{v}+\Delta_{s},\;\begin{array}{cc} W_{slot}^{\min} & =W_{v}+\Delta_{left}+\Delta_{right}\\ & =\left\{ \begin{array}{l} 2\left(\left\lceil \frac{W_{v}}{2(R_{\min}-\sqrt{R_{\min}^{2}-{\left(\Delta_{s}/2\right)}^{2}})}\right\rceil -\frac{1}{2}\right)(R_{\min}-\sqrt{R_{\min}^{2}-{\left(\Delta_{s}/2\right)}^{2}})+\frac{W_{v}\sqrt{R_{\min}^{2}-{\left(\Delta_{s}/2\right)}^{2}}}{2R_{\min}}\\ +\frac{\left(L_{v}-L_{bh}\right)\Delta_{s}}{2R_{\min}}-\frac{W_{v}}{2}\;\mathrm{Situation}1\\ 2\left(\left\lceil \frac{W_{v}}{2(R_{\min}-\sqrt{R_{\min}^{2}-{\left(\Delta_{s}/2\right)}^{2}})}\right\rceil -\frac{1}{2}\right)(R_{\min}-\sqrt{R_{\min}^{2}-{\left(\Delta_{s}/2\right)}^{2}})+\frac{W_{v}\sqrt{R_{\min}^{2}-{\left(\Delta_{s}/2\right)}^{2}}}{2R_{\min}}\\ +\max(\frac{\left(L_{v}-L_{bh}\right)\Delta_{s}}{2R_{\min}}-2(R_{\min}-\sqrt{R_{\min}^{2}-{\left(\Delta_{s}/2\right)}^{2}}),\frac{L_{bh}\Delta_{s}}{2R_{\min}})-\frac{W_{v}}{2}\;\mathrm{Situation}2 \end{array}\right.\\ W_{exter}^{\min} & =W_{v}+\Delta_{exter}\\ & =\max(\frac{\left(L_{v}-L_{bh}\right)\Delta_{s}}{2R_{\min}}-3(R_{\min}-\sqrt{R_{\min}^{2}-{\left(\Delta_{s}/2\right)}^{2}}),\frac{L_{bh}\Delta_{s}}{2R_{\min}}-(R_{\min}-\sqrt{R_{\min}^{2}-{\left(\Delta_{s}/2\right)}^{2}})).\\ & \;+\frac{W_{v}\sqrt{R_{\min}^{2}-{\left(\Delta_{s}/2\right)}^{2}}}{2R_{\min}}+\frac{W_{v}}{2} \end{array}$$

*where* $L_{slot}^{\min}$*,* $W_{slot}^{\min}$ *are the minimum length and width of the internal parking space,* $L_{exter}^{\min}$*,* $W_{exter}^{\min}$ *are the minimum length and width of the external parking space respectively;* $\Delta_{s}$ *is the minimum longitudinal displacement brought about by an S-shaped effective movement (trajectory composed by two symmetric circle arcs), which is determined by the vehicle mechanical properties only. As shown in Figure 2b,* $\Delta_{left}$ *is the lateral distance between the vehicle and the left boundary of the parking slot after the parking maneuver is completed;* $\Delta_{right}$ *represents the lateral distance between the right boundary of the vehicle and the furthest position on the right reached during the whole parking maneuver. And* $\Delta_{right}$ *represents the lateral distance between the left boundary of the vehicle at the initial position and the furthest position on the left reached during the whole parking maneuver. The proof of Theorem 1 will be given in the rest of this section.*

**Proof** **of** **Theorem** **1.**  

Calculation of $L_{slot}^{\min}$ and $L_{exter}^{\min}$

In order to facilitate the parking maneuver, vehicle needs to perform S-shaped forward and backward movement alternately. Each forward movement produces a longitudinal displacement of $\Delta_{s}$, and the subsequent backward movement brings the vehicle back to the initial longitudinal position. Hence, the maximum longitudinal displacement is just $\Delta_{s}$ during the whole parking maneuver. Then the minimum lengths of the internal and external space required for parking can both be given by

(6) $$L_{slot}^{\min}=L_{exter}^{\min}=L_{v}+\Delta_{s}$$

Calculation of $W_{slot}^{\min}$ and $W_{exter}^{\min}$

The minimum width of the parking slot (i.e., internal space) is equal to the width of the vehicle plus the left redundancy $\Delta_{left}$ and the right redundancy $\Delta_{right}$. The left redundancy comes from the fact that the n-times lateral displacement cannot ensure that the left sides of the vehicle and the parking slot are just overlapped after parking. $\Delta_{left}$ can be expressed as $\Delta_{left}=nd_{once}-W_{v}$, where $d_{once}$ is the lateral displacement caused by an S-shaped movement and n can be given by $n=\left\lceil W_{v}/d_{once}\right\rceil$. The right redundancy $\Delta_{right}$ is related to the furthest position on the right that the vehicle can reach during the whole parking maneuver.

Calculation of $\Delta_{left}$

Since the vehicle has symmetrical characteristics and the maximum left and right steering angles are equal, so when moving with the maximum steering angle, the turning radius of each circle arcs are the same and the minimum value $R_{\min}$, and the corresponding center angle $\theta_{m}$ is also the same. The lateral displacement $d_{once}$ brought by each S-shaped movement is constant, so the number of trials can be calculated after clarifying the total lateral displacement d required for parking. We have assumed that the vehicle is parked next to the parking slot at the initial position, then there is $d=W_{v}$. The reference point of the vehicle at the initial position is recorded as $P_{init}$, the tangent point is C and the rotation center during the first circle arc is recorded as $O_{r}$, the coordinate system is established as shown in Figure 2. Analyzing the geometric relations in triangle $P_{init}O_{r}C$, it results:

(7) $$d_{once}=2(R_{\min}-\sqrt{R_{\min}^{2}-{\left(\Delta_{s}/2\right)}^{2}})$$

(8) $$\left\{ \begin{array}{l} \sin\theta_{m}=\frac{\Delta_{s}}{2R_{\min}}\\ \cos\theta_{m}=\frac{R_{\min}-d_{once}/2}{R_{\min}}=\frac{\sqrt{R_{\min}^{2}-{\left(\Delta_{s}/2\right)}^{2}}}{R_{\min}} \end{array}\right.$$

Then the number of trials is

(9) $$n=\left\lceil \frac{W_{v}}{2(R_{\min}-\sqrt{R_{\min}^{2}-{\left(\Delta_{s}/2\right)}^{2}})}\right\rceil$$

Thus, there is

(10) $$\Delta_{left}=2\left\lceil \frac{W_{v}}{2(R_{\min}-\sqrt{R_{\min}^{2}-{\left(\Delta_{s}/2\right)}^{2}})}\right\rceil (R_{\min}-\sqrt{R_{\min}^{2}-{\left(\Delta_{s}/2\right)}^{2}})-W_{v}$$

Calculation of $\Delta_{right}$

**Figure 3:**
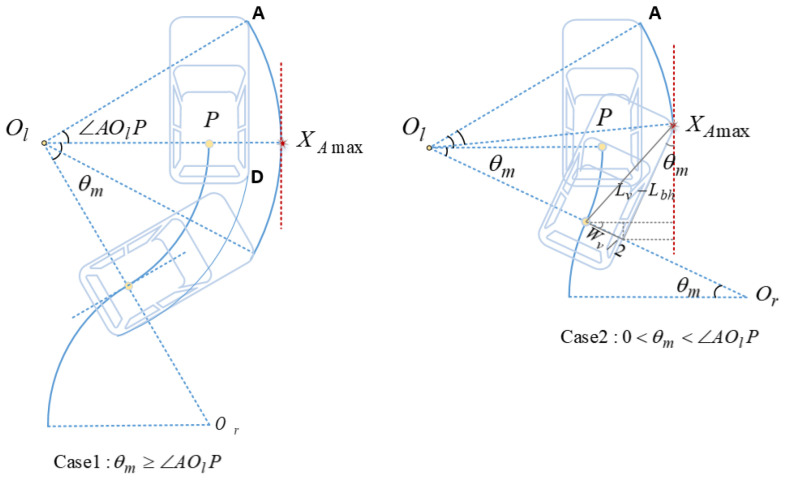
Two cases of S-shaped forward movement.

The right redundancy is related to the furthest position on the right that can be reached during the parking maneuver. Therefore the trajectory of the bounding rectangle of the vehicle needs to be considered. When the midpoint of the vehicle’s rear axle (reference point) moves from the tangent point to the upper end position, the turning radius of A (the right front corner of the vehicle) is the largest of all points on the vehicle and thus the furthest position can be reached by A. Therefore, as shown in Figure 3, when the vehicle makes an S-shaped forward movement, A is selected as the analysis target to determine the furthest position that can be reached during the maneuver. Let $\theta_{m}$ be the central angle of a circle arc; let $∠AO_{l}P$ be the acute angle between line $\bar{O_{l}P}$ and $\bar{AO_{l}}$. Then the exact location of the furthest position needs to be considered in two cases.

Case 1 $\theta_{m}\geq ∠AO_{l}P$: Considering the backward movement from the upper end position $P_{end}(X_{end},Y_{end})$ to the tangent point, it can be obtained from the analysis of the characteristics of the circle in plane geometry that the right-side furthest position that A can reach is the intersection of the line $\bar{O_{l}P}$ and the circle $C\langle O_{l},R_{O_{l}A}\rangle$ with $O_{l}$ and $R_{O_{l}A}$ being the center and radius respectively. As shown in the left diagram of Figure 3, The horizontal coordinates of the farthest point $X_{A\max}$ can be obtained from analytic geometry:

(11) $$X_{A\max}=X_{end}-R_{\min}+R_{O_{l}A}$$

with

(12) $$R_{O_{l}A}=\sqrt{(R_{\min}+W_{v}/2{)}^{2}+{\left(L_{v}-L_{bh}\right)}^{2}}$$

Case 2 $0<\theta_{m}<∠AO_{l}P$: During the movement from the upper end position to the tangent point, A moves in a circular arc around the left rotation center $O_{l}$, and after passing the tangent point, A moves in a circular arc around the right rotation center $O_{r}$. As shown in the right diagram of Figure 3, since A cannot reach the ideal farthest position in geometric relationship, accordingly when the reference point is located at the tangent point $\left(X_{c},Y_{c}\right)$, the location of A is the farthest position the vehicle can reach during the maneuver. $X_{A\max}$ can be obtained from analytic geometry:

(13) $$X_{A\max}=X_{c}+\frac{W_{v}}{2}*\cos\theta_{m}+\left(L-L_{bh}\right)*\sin\theta_{m}=X_{c}+\frac{W_{v}\sqrt{R_{\min}^{2}-{\left(\Delta_{s}/2\right)}^{2}}}{2R_{\min}}+\frac{\left(L-L_{bh}\right)\Delta_{s}}{2R_{\min}}.$$

When the vehicle makes an S-shaped backward movement, the right rear corner of the vehicle, denoted as D, has the largest turning radius. Therefore, the furthest position can be reached by D during the maneuver. The exact location of the furthest position needs to be considered in two cases as well.

**Figure 4:**
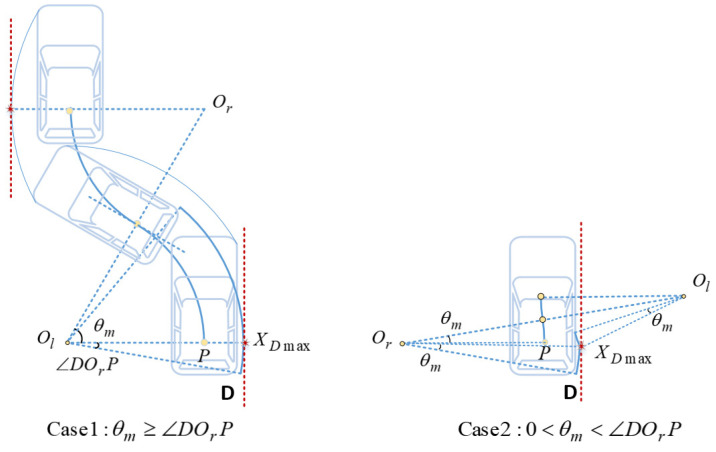
Two cases of S-shaped backward movement.

Case 1 $\theta_{m}\geq ∠DO_{r}P$: Consider reversing the process of backward movement, as shown in the left-hand diagram of Figure 4. From the lower end to the tangent point, the furthest position on the right side that D can reach is the intersection of the line $\bar{O_{l}P}$ and the circle $C\langle O_{l},R_{O_{l}D}\rangle$. The horizontal coordinates of the farthest point $X_{\max}$ can be obtained from analytic geometry:

(14) $$X_{D\max}=X_{end}-R_{\min}+R_{O_{l}D}$$

where

(15) $$R_{O_{l}D}=\sqrt{(R_{\min}+W_{v}/2{)}^{2}+L_{bh}{}^{2}}$$

Case 2 $0<\theta_{m}<∠DO_{r}P$: As shown in the right diagram of Figure 4, D cannot reach the ideal farthest position in geometric relationship. Therefore when the reference point is located at the tangent point $\left(X_{c},Y_{c}\right)$, the location of D is the farthest position the vehicle can reach during the parking maneuver. $X_{D\max}$ can be obtained from analytic geometry:

(16) $$X_{D\max}=X_{c}+\frac{W_{v}}{2}*\cos\theta_{m}+L_{bh}*\sin\theta_{m}=X_{c}+\frac{W_{v}\sqrt{R_{\min}^{2}-{\left(\Delta_{s}/2\right)}^{2}}}{2R_{\min}}+\frac{L_{bh}\Delta_{s}}{2R_{\min}}.$$

**Figure 5:**
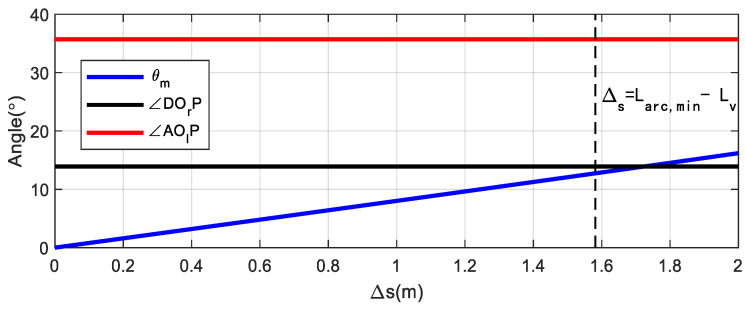
The trend of maximum central angle.

In this section, n-trial parking is needed for space saving, so the minimum slot length must be smaller than $L_{arc,\min}$, which is the slot length required for one-trial parking based on circle arcs mentioned in [14]. For the vehicle model mentioned in Table 1, there is $L_{arc,\min}=5.817$, so the upper bound of $\Delta_{s}$ should be less than $L_{arc,\min}-L_{v}=1.582\;m$. The trend of $\theta_{m}$ is shown in Figure 5, when $\Delta_{s}=1.582\;m$, there are $\theta_{m}=12.74{}^{\circ}$, $∠DO_{r}P=12.92{}^{\circ}$, $∠AO_{l}P=35.70{}^{\circ}$, and when $\Delta_{s}<L_{arc,\min}-L_{v}$, there is $0<\theta_{m}<∠DO_{r}P<∠AO_{l}P$ absolutely, so the subsequent analysis about the left and right redundancy in this section are both based on case 2.

The last issue to be discussed is whether the vehicle is at the top or the bottom of the parking slot after the parking maneuver is completed, which would result in different $\Delta_{right}$, shown as follows.

1. Situation 1: The vehicle is parked at the top of the slot. In this case, the trajectory of the vehicle shows that $\Delta_{right}$ is only related to the furthest position reached by the last S-shaped forward movement. It can be concluded from the analytical geometry and Equation (13) that
  (17) $$\Delta_{right,up}=X_{A\max}-(X_{c}+\frac{d_{once}}{2}+\frac{W_{v}}{2})=\frac{W\sqrt{R_{\min}^{2}-{\left(\Delta_{s}/2\right)}^{2}}}{2R_{\min}}+\frac{\left(L_{v}-L_{bh}\right)\Delta_{s}}{2R_{\min}}-\frac{d_{once}}{2}-\frac{W_{v}}{2}$$
2. Situation 2: The vehicle is parked at the bottom of the slot. In this case, $\Delta_{right}$ is determined by the farther position that can be reached by the penultimate S-shaped forward movement and the last backward movement. From Equations (13) and (16), there is
  (18) $$\begin{array}{cc} \Delta_{right,down} & =\max(X_{A\max}-(X_{c}+\frac{3d_{once}}{2}+\frac{W_{v}}{2}),X_{D\max}-(X_{c}+\frac{d_{once}}{2}+\frac{W_{v}}{2}))\\ & =\frac{W\sqrt{R_{\min}^{2}-{\left(\Delta_{s}/2\right)}^{2}}}{2R_{\min}}+\max(\frac{\left(L-L_{bh}\right)\Delta_{s}}{2R_{\min}}-\frac{3d_{once}}{2},\frac{L_{bh}\Delta_{s}}{2R_{\min}}-\frac{d_{once}}{2})-\frac{W_{v}}{2} \end{array}$$

Calculation of $\Delta_{exter}$

The first two trials of the parking maneuver are the same as the last two mentioned in Situation 2 in the previous subsection, and the external redundancy required for parking is only related to the farthest left position reached by the vehicle’s left front corner B and left rear corner C. Same analysis as in Situation 2, from symmetry it follows that:

(19) $$\Delta_{exter}=\Delta_{right,down}$$

Final Results

From Equations (7), (10), (17) and (18), the minimum width of internal parking space (parking slot) can be calculated by the following formula:

(20) $$W_{slot}^{\min}=\left\{ \begin{array}{l} 2\left(\left\lceil \frac{W_{v}}{2(R_{\min}-\sqrt{R_{\min}^{2}-{\left(\Delta_{s}/2\right)}^{2}})}\right\rceil -\frac{1}{2}\right)(R_{\min}-\sqrt{R_{\min}^{2}-{\left(\Delta_{s}/2\right)}^{2}})+\frac{W_{v}\sqrt{R_{\min}^{2}-{\left(\Delta_{s}/2\right)}^{2}}}{2R_{\min}}\\ +\frac{\left(L_{v}-L_{bh}\right)\Delta_{s}}{2R_{\min}}-\frac{W_{v}}{2}\;\mathrm{Situation}1\\ 2\left(\left\lceil \frac{W_{v}}{2(R_{\min}-\sqrt{R_{\min}^{2}-{\left(\Delta_{s}/2\right)}^{2}})}\right\rceil -\frac{1}{2}\right)(R_{\min}-\sqrt{R_{\min}^{2}-{\left(\Delta_{s}/2\right)}^{2}})+\frac{W_{v}\sqrt{R_{\min}^{2}-{\left(\Delta_{s}/2\right)}^{2}}}{2R_{\min}}\\ +\max(\frac{\left(L_{v}-L_{bh}\right)\Delta_{s}}{2R_{\min}}-2(R_{\min}-\sqrt{R_{\min}^{2}-{\left(\Delta_{s}/2\right)}^{2}}),\frac{L_{bh}\Delta_{s}}{2R_{\min}})-\frac{W_{v}}{2}\;\mathrm{Situation}2 \end{array}\right.$$

From Equations (7), (10), (18) and (19), the minimum width of external parking space can be calculated by the following formula:

(21) $$\begin{array}{cc} W_{exter}^{\min}= & \max\left(\frac{\left(L_{v}-L_{bh}\right)\Delta_{s}}{2R_{\min}}-3(R_{\min}-\sqrt{R_{\min}^{2}-{\left(\Delta_{s}/2\right)}^{2}}),\frac{L_{bh}\Delta_{s}}{2R_{\min}}-(R_{\min}-\sqrt{R_{\min}^{2}-{\left(\Delta_{s}/2\right)}^{2}})\right)\\ & +\frac{W_{v}\sqrt{R_{\min}^{2}-{\left(\Delta_{s}/2\right)}^{2}}}{2R_{\min}}+\frac{W_{v}}{2} \end{array}$$

The proof of Theorem 1 is completed. □

### Discussions

**Figure 6:**
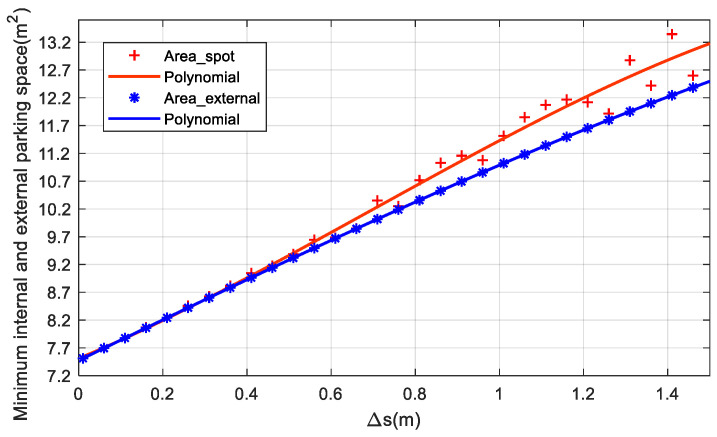
Variation of minimal internal and external parking space.

From Table 2 and Figure 6, it can be seen that when $\Delta_{s}$ tends to 0, the minimum internal and external parking space tends to the size of the bounding rectangle of the vehicle. With the increase of $\Delta_{s}$, the minimum external parking space shows a linear trend of growth, while the rise of the minimum internal parking space is relatively nonlinear, but it shows an overall upward trend.

In the practical application scenario, the parking space calculated by Equation (5) is the lower limit of space required for parking, which is also the guideline for parking space detection. The space less than this lower limit will not be considered as feasible parking space and will be ignored by the detection system. When the space detected by the system is relatively large, $\Delta_{s}$ can be flexibly replaced by $d_{left}$ according to the detected available space ($d_{left}$ is the difference between the actual detected parking slot length and the vehicle length), so as to complete the parking task with less parking trials and improve the driving comfort. In addition, if the requirement for external parking space is low, the first parking trajectory can also be flexibly changed to the one mentioned in the next section, thus further reducing the number of trials.

**Figure 7:**
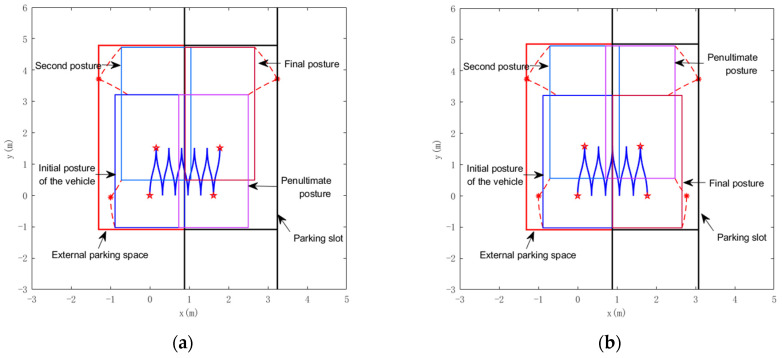
Simulation of the parking for two situations: (**a**) situation1; (**b**) situation 2.

The simulation on Matlab of the vehicle in two parking situations is represented in Figure 7. When the number of trials is odd, the vehicle is parked at the top of the parking slot, and when it is even, the vehicle is parked at the bottom of the parking slot. The blue solid line is the trajectory of the reference point of the vehicle, and the red dashed line is the trajectory of the points on the vehicle that may affect the size of the parking space, while the simulation parameters are given in Table 1.

## 4. Minimum Space for One-Trial Parking Based on Circle Arcs

The minimum parking space required for automatic parallel parking based on circle arcs has been given in Section 3, and the conclusion tends to be an idealized derivation with unbounded number of trials. Considering the minimum time and fuel consumption, as well as the optimal comfort for passengers, this section will give the parking space required for the classic one-trial parking based on admissible circle arcs (one-trial parking, i.e., park the car without longitudinal velocity sign change). In this method, the “simplified” kinematic model with only three configuration parameters for the vehicle is used, and the path is composed by two circular arcs connected by a tangent point. The minimum length $L_{\min}$ and width $W_{\min}$ of the parking slot has been given in [19], which will not be discussed here. This section focuses on the minimum external parking space required by the one-trial circular arc parking, which can be represented by a simplified rectangular area.

**Theorem** **2.** *The minimum length and width of external parking space required for one-trial parking based on admissible circular arcs can be obtained by the following formula:*

(22) $$\begin{array}{cc} L_{exter}^{\min} & =2R_{\min}\sin\theta_{S_{\min}}+L_{v}-L_{bh}-\\ & \min\left(\begin{array}{l} 2R_{\min}\sin\theta_{S_{\min}}-\sqrt{R_{O_{r}C}{}^{2}-{\left(-W_{v}/2-W_{left}-R_{\min}(2\cos\theta_{S_{\min}}-1)\right)}^{2}}\\ ,\sqrt{R_{lw}{}^{2}-{\left(-W_{v}/2-W_{left}+R_{\min}\right)}^{2}} \end{array}\right) \end{array}$$

(23) $$W_{exter}^{\min}=R_{\min}(1-2\cos\theta_{S_{\min}})+R_{O_{r}B}-W_{v}/2-W_{left}$$

*where* $W_{left}$ *denotes the lateral distance between the vehicle and the left boundary of the parking slot at the end of the parking, which can be taken according to the actual application requirements, and when it is regarded as 0, the size of the external parking space is only depends on the characteristics of the vehicle.* $\theta_{S_{\min}}$ *denotes the minimum central angle of the arc to meet the vehicle exit collision-free conditions.* $R_{O_{r}B}$*,* $R_{O_{r}C}$ *and* $R_{lw}$ *are the turning radius of point* C*, point* B *and the left rear wheel of the vehicle when the instantaneous center of rotation is* $O_{r}$*, respectively, and by symmetry,* $R_{O_{r}B}=R_{O_{l}A}$*,* $R_{O_{r}C}=R_{O_{l}D}$ *. The proof of Theorem 2 will be given in the rest of this section.*

**Proof** **of** **Theorem** **2.**  

Determination of Parameter $\theta_{S_{\min}}$

The parking maneuver is considered in reverse, two conditions need to be meet for the vehicle does not collide with the parking space in the process of exit the spot: (1.) During the first circle arc, the right front and rear corner of the vehicle have, respectively, no collision with the front and lateral obstacles of the parking slot. The minimum length and width of the parking slot can be geometrically calculated by this condition. (2.) During the second circle arc, the right rear wheel of the vehicle has no collision with the surrounding obstacles of the parking slot and other parked vehicles. Under this condition, the minimum central angle of the arc $\theta_{S_{\min}}$ corresponding to the collision-free condition can be calculated geometrically, provided that $W_{left}$ and vehicle characteristics are known.

**Figure 8:**
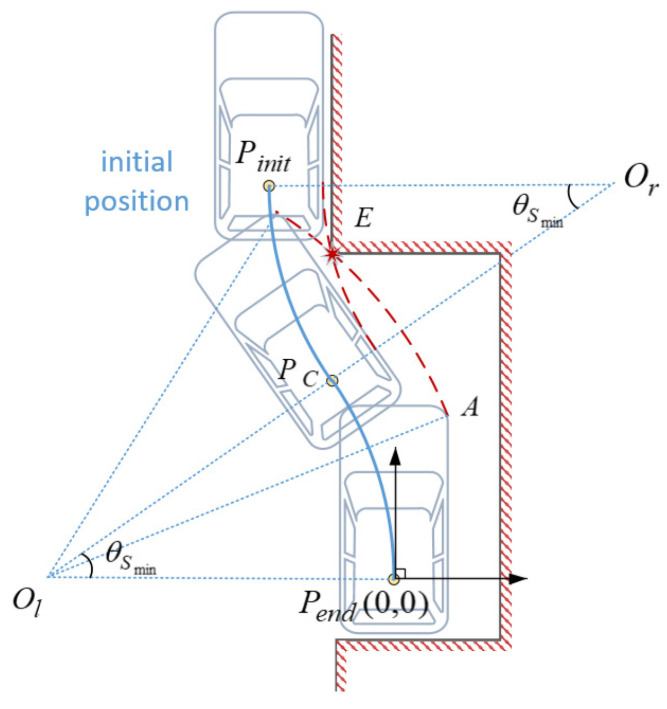
Parking in one trial based on circle arcs.

The coordinate system is established as shown in Figure 8, At the end of the parking, the coordinate of the reference point of the vehicle is $P_{end}(0,0)$, so the coordinates of the tangent point and the left and right rotation centers can be obtained from the geometric relationship:

(24) $$P_{c}(R_{\min}(\cos\theta_{S}-1),R_{\min}\sin\theta_{S})\;P_{init}(2R_{\min}(\cos\theta_{S}-1),2R_{\min}\sin\theta_{S})\;O_{l}\left(X_{O_{l}},Y_{O_{l}}\right)=(-R_{\min},0)\;O_{r}\left(X_{O_{r}},Y_{O_{r}}\right)=(R_{\min}(2\cos\theta_{S}-1),2R_{\min}\sin\theta_{S}).$$

The trajectory equation of the right rear wheel of the vehicle during the second circle arc is:

(25) $$(x-X_{O_{r}}{)}^{2}+(y-Y_{O_{r}}{)}^{2}=R_{rw}{}^{2}$$

where

(26) $$R_{rw}=R_{\min}-W_{v}/2.$$

Under the extreme conditions, $\theta_{S}=\theta_{S_{\min}}$, the right rear wheel of the vehicle should pass exactly the upper left corner $E(-W_{v}/2-W_{left},L_{slot}-L_{bh})$ of the parking space during the movement, where, $L_{slot}=L_{bh}+\sqrt{R_{O_{l}A}^{2}-{\left(R_{\min}-W_{v}/2-W_{left}\right)}^{2}}$ is the length of the parking slot, substituting this into above trajectory Equation (25) yields $\theta_{S_{\min}}$, as shown in Table 3, which gives the specific values for the $\theta_{S_{\min}}$ in different $W_{left}$ conditions.

Calculation of $W_{exter}^{\min}$

When the center angle is $\theta_{S_{\min}}$ and the radius of both circle arcs is the minimum value, the external space required for parking is minimized, which can be represented by a simplified rectangular area, and the size of it is recorded as $Area_{out}$. For regular small cars, there are $pi/2>\theta_{S_{\min}}>∠AO_{l}P>∠DO_{r}P$ (see Table 3). Therefore, the analysis of the farthest position in this section is all based on the case1 model introduced in the previous section. The right boundary of the rectangular area representing the external parking space is the left boundary of the parking slot, and its horizontal coordinate is $-W_{v}/2-W_{left}$. The left boundary of the rectangular area is determined by the farthest left position reached by the left front corner B during the parking process, denoted as $X_{Left}$ (as can we see in Figure 4).

(27) $$X_{Left}=X_{O_{r}}-R_{O_{r}B}=R_{\min}(2\cos\theta_{S_{\min}}-1)-R_{O_{r}B}$$

where $R_{O_{r}B}=R_{O_{l}A}=\sqrt{(R_{\min}+W_{v}/2{)}^{2}+{\left(L_{v}-L_{bh}\right)}^{2}}$.

Thus, the minimum width of the external parking space is:

(28) $$W_{exter}^{\min}=R_{\min}(1-2\cos\theta_{S_{\min}})+R_{O_{r}B}-W_{v}/2-W_{left}$$

Calculation of $L_{exter}^{\min}$

The upper boundary of the rectangular area is determined by the highest position reached by the header of the vehicle during the parking process, noted as $Y_{\max}$. The lower boundary of the rectangular area is determined by the lowermost intersection of the outer contour of the vehicle and the left boundary of the parking slot during the whole parking maneuver, that is, the lower position of the intersection of the left rear corner trajectory of the vehicle during the first circle arc and the left rear wheel trajectory of the vehicle during the second circle arc with the left boundary of the parking slot, noted as $Y_{\mathrm{intersection}}$. As a result, $Y_{\max}$, $Y_{\mathrm{intersection}}$ can be obtained as follows:

(29) $$Y_{\max}=Y_{O_{r}}+L_{v}-L_{bh}=2R_{\min}\sin\theta_{S_{\min}}+L_{v}-L_{bh}$$

(30) $$Y_{\mathrm{intersection}}=\min\left(Y_{O_{r}}-\sqrt{R_{O_{r}C}{}^{2}-{\left(-W_{v}/2-W_{left}-X_{Or}\right)}^{2}},\sqrt{R_{lw}{}^{2}-{\left(-W_{v}/2-W_{left}-X_{O_{l}}\right)}^{2}}\right)$$

So the minimum length of the external parking space is:

(31) $$\begin{array}{l} L_{exter}^{\min}=Y_{\max}-Y_{\mathrm{intersection}}\\ =2R_{\min}\sin\theta_{S_{\min}}+L_{v}-L_{bh}-\\ \min\left(2R_{\min}\sin\theta_{S_{\min}}-\sqrt{R_{O_{r}C}{}^{2}-{\left(-W_{v}/2-W_{left}-R_{\min}(2\cos\theta_{S_{\min}}-1)\right)}^{2}},\sqrt{R_{lw}{}^{2}-{\left(-W_{v}/2-W_{left}+R_{\min}\right)}^{2}}\right) \end{array}$$

where, $R_{O_{r}C}=R_{O_{l}D}=\sqrt{(R_{\min}+W_{v}/2{)}^{2}+L_{bh}{}^{2}}$ and $R_{lw}=R_{\min}-W_{v}/2$.

The proof of Theorem 2 is completed. □

### Discussions

**Figure 9:**
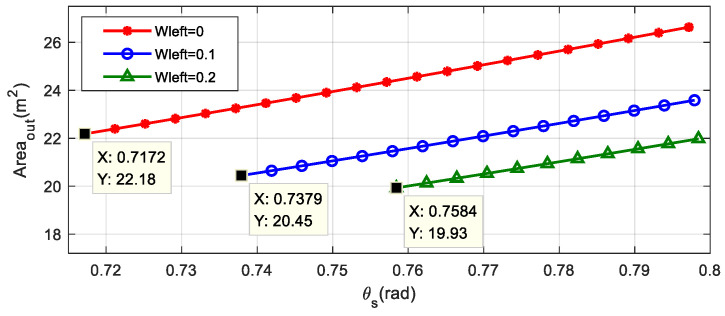
Variation of the minimal external parking space for different $W_{left}$.

It is illustrated in Figure 9 that size of the external parking space under different values of $W_{left}$ are growing with the increase of the central angle $\theta_{S}$. As a consequence, the external space required for parking is the smallest when $\theta_{S}=\theta_{S_{\min}}$. When $W_{left}=0$, the minimal external parking space is only related to the characteristics and mechanical properties of the vehicle, which can be widely extended to different scenarios.

In a real application scenario, if the lateral distance between the vehicle and the parking space at the initial position is far, the turning radius of the first arc can be appropriately increased to meet the actual parking demand, and of course, the required external parking space will also be increased.

**Figure 10:**
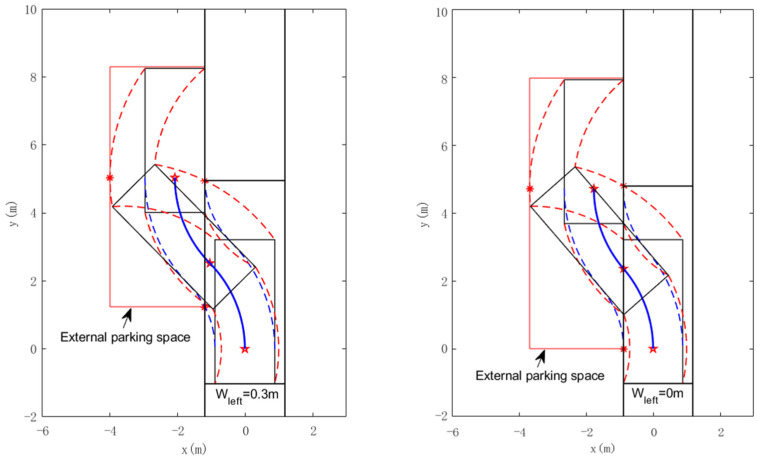
Simulation of the parking for two different $W_{left}$.

The parking trajectory and the bounding rectangle of external parking space (red rectangular area) are shown in Figure 10, when $W_{left}=0.3$, the external parking space is smaller, but the required space for parking slot is larger, when $W_{left}=0$, the situation is the opposite. In actual application scenarios, the automatic parking system can choose $W_{left}$ flexibly according to the scene information to complete the parking task at the least possible cost.

## 5. Minimum Space for One-Trial Parking Based on Continues-Curvature Curve

In the previous section, the minimum space required for parking in one trial based on admissible circle arcs is discussed. The parking space required by that method is almost the smallest in all one-trial parking methods. However, the curvature of this type of path is discontinuous and discontinuities occur at every transition between arcs with an opposite direction of rotation. The vehicle have to stop at every discontinuous point to reorient its front wheels. This process not only increases the total parking time, but also causes wear on the steering column and wheels. Parking based on continuous-curvature curve is a good choice to solve these problems. Considering the kinematics model of the vehicle in Section 2, a real car can well track the path with continuous curvature. The path has upper-bounded curvature and upper-bounded curvature derivative, which come from the fact that the vehicle steering angle and angular velocity have upper bounds. So in this section, clothoid is chosen to achieve the smooth transition of curvature, which is a parametric curve whose curvature is a polynomial function of its arc length and can satisfy all the three constraints mentioned above. As a matter of fact, the continuous-curvature curve (referred as CC curve) discussed in this section is made up of circular arcs and clothoids. Besides, in the same manner as the analysis in the previous section, the process of parking into the slot is considered in reverse and the path is constructed from the end of the parking.

Continuous-curvature path achieves smooth transition at the expense of more parking space, which greatly improves the comfort of driving. Compared with parking based on circle arcs, what is the increment of parking space, and what is the minimum internal and external space required for one-trial parking based on continues-curvature curve? These questions will be answered in this section.

**Theorem** **3.** *The lower and upper bounds of the minimum length and width of internal parking space required for one-trial parking based on continuous-curvature curve can be obtained by the following formula:*

(32) $$\left\{ \begin{array}{l} L_{slot}^{\min}=L_{bh}+\Delta_{1}+\sqrt{R_{A\min}^{2}-{\left(\Delta_{2}+R_{\min}-W_{v}/2\right)}^{2}}\\ L_{slot}^{\max}=L_{bh}+\Delta_{1}+\sqrt{R_{A\max}^{2}-{\left(\Delta_{2}+R_{\min}-W_{v}/2\right)}^{2}} \end{array}\right.\left\{ \begin{array}{l} W_{slot}^{\min}=W_{v}/2+R_{D\min}-(R_{\min}+\Delta_{2})\\ W_{slot}^{\max}=W_{v}/2+R_{D\max}-(R_{\min}+\Delta_{2}) \end{array}\right.$$

*the lower and upper bounds of the minimum length and width of external parking space:*

(33) $$L_{exter}=2R_{\min}\sin\theta_{contact}+2(\Delta_{1}+\Delta_{3})+L_{v}-L_{bh}\;\left\{ \begin{array}{l} W_{exter}^{\min}=2R_{\min}(1-\cos\theta_{contact})+\Delta_{2}+2\Delta_{4}+R_{B\min}-R_{\min}-W_{v}/2\\ W_{exter}^{\max}=2R_{\min}(1-\cos\theta_{contact})+\Delta_{2}+2\Delta_{4}+R_{B\max}-R_{\min}-W_{v}/2 \end{array}\right.$$

*where,* $\Delta_{1}$ *and* $\Delta_{3}$ *are the increments in the horizontal direction of the continues-curvature path with respect to the circular arc path,* $\Delta_{2}$ *and* $\Delta_{4}$ *are vertical increments,* $\theta_{contact}$ *is the orientation angle at the contact point.* $R_{A\min},R_{A\max}$ *are the lower and upper limit of the minimum turning radius of* A *respectively, and the same analysis for* $R_{B},R_{D}$*. The proof of Theorem 3 will be given in the rest of this section.*

### 5.1. General Properties of Clothoid

A clothoid is a curve whose curvature varies linearly with its arc length:

(34) k(l)=σl+k(0)

where l is the length of the curve from the starting point to the current point, and σ is the sharpness of the clothoid. The longer the length of the clothoid, the better the smoothing effect of the curve, but too long clothoid will lead to a long parking path and increase the requirement of parking space. Hence, to minimize the parking space, we take the maximum value of the steering angle and steering angular velocity, so that the length of the clothoid is as short as possible. The configuration of the reference point of the vehicle on the clothoid is defined as $q_{i}=(x_{i},y_{i},\theta_{i},k_{i})$, the angle between the orientation of $q_{i}$ and the tangent to the equivalent center of circle is denoted as $\beta_{i}$. In the initial position, the curvature is 0, and the turning radius is ∞, $\beta_{\max}$ recorded as $\beta_{i}$ with maximum value. Subsequently the curvature gradually increases at a constant rate until it to the maximum value $k_{\max}$ when the radius reaches a minimum value $R_{\min}$, $\beta_{i}$ decreasing to 0 and the clothoid arc ended at this point. Thereafter, if the path continues at maximum curvature, the trajectory is transformed into a circular arc of constant curvature.

The whole continuous-curvature path (referred as CC path) consists of two symmetric segments about the center of the contact point. The First segment of the path is made up of three parts: (1) a clothoid arc of sharpness $\sigma =\sigma_{\max}$ whose curvature varies from 0 to $k_{\max}$; (2) a circular arc of radius $R_{\min}=k_{\max}^{-1}$; (3) a clothoid arc of sharpness −σ whose curvature varies from $k_{\max}$ to 0. At the end of this segment, the curvature is 0, and the orientation of the vehicle is $\theta_{contact}$. The next segment is the inversion of this segment, where the path curvature varies continuously linearly up from 0 and then gradually back to 0. The vehicle track along the continues-curvature path is small-time controllable [31], And the set of configurations reachable from any configuration q before a time t contains a neighborhood of q for any *t*. When the vehicle velocity is constant, the configuration $q_{t}$ of the vehicle at time *t* is:

(35) $$q_{t}=\left\{ \begin{array}{l} x_{t}=\sqrt{\frac{\pi }{\sigma_{\max}}}\int_{0}^{\sqrt{\frac{\sigma_{\max}}{\pi }}\cdot vt}\cos\frac{t^{2}}{2}dt\\ y_{t}=\sqrt{\frac{\pi }{\sigma_{\max}}}\int_{0}^{\sqrt{\frac{\sigma_{\max}}{\pi }}\cdot vt}\sin\frac{t^{2}}{2}dt\\ \theta_{t}=\frac{\sigma_{\max}v^{2}t^{2}}{2}\\ k_{t}=\sigma_{\max}vt \end{array}\right.t\in [0,\frac{\delta_{\max}}{v_{\delta }}]$$

The whole length of clothoid arc $L_{s}=k_{\max}/\sigma_{\max}=v\cdot t_{\min}$(v is the longitudinal velocity of the vehicle), and the steering angle of the vehicle are turning at a constant maximum angular velocity to $\delta_{\max}$, so there are $t_{\min}=\delta_{\max}/v_{\delta }$ Combining these equations, the following relationship about the sharpness of clothoid can be deduced:

(36) $$\sigma_{\max}=\frac{k_{\max}}{L_{s}}=\frac{v_{\delta }}{v\cdot \delta_{\max}\cdot R_{\min}}$$

In order to ensure the existence of circular arc segments in the path, the orientation angle at the contact point $\theta_{contact}$ needs to satisfy certain conditions, otherwise, the planned path will become a path consisting entirely of clothoid arcs. The variation of vehicle orientation after a clothoid arc is $\theta_{clo}=k_{\max}^{2}/2\sigma_{\max}$ [28]. From symmetry, the following conditions need to be satisfied in the presence of a circular arc segment:

(37) $$\theta_{contact}\geq 2\theta_{clo}=\frac{k_{\max}^{2}}{\sigma_{\max}}$$

**Figure 11:**
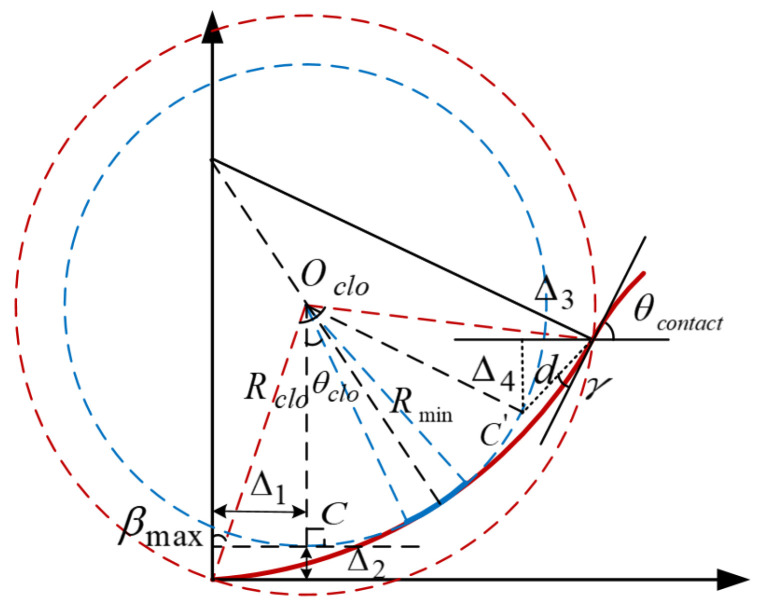
Continuous-curvature turns.

The coordinate system is established as shown in Figure 11, the configuration of the vehicle at the end of the parking is $q_{0}=(0,0,0,0)$, hence the coordinates of the end of the first clothoid arc and the center of the circular arc $O_{clo}$ are:

(38) $$\left\{ \begin{array}{l} x_{1}=\sqrt{\pi /\sigma_{\max}}FrC(\sqrt{\frac{k_{\max}^{2}}{\sigma_{\max}}})\\ y_{1}=\sqrt{\pi /\sigma_{\max}}FrS(\sqrt{\frac{k_{\max}^{2}}{\sigma_{\max}}}) \end{array}\right.\left\{ \begin{array}{l} x_{clo}=\sqrt{\pi /\sigma_{\max}}FrC(\sqrt{\frac{k_{\max}^{2}}{\sigma_{\max}}})-R_{\min}\sin(\theta_{clo})\\ y_{clo}=\sqrt{\pi /\sigma_{\max}}FrS(\sqrt{\frac{k_{\max}^{2}}{\sigma_{\max}}})+R_{\min}\cos(\theta_{clo}) \end{array}\right.$$

with FrC and FrS, the Fresnel integrals:

(39) $$FrC(x)=\int_{0}^{x}\cos\frac{\pi }{2}u^{2}du,\;FrS(x)=\int_{0}^{x}\sin\frac{\pi }{2}u^{2}du.\;$$

The distance from the beginning of the clothoid segment to the center $O_{clo}$ is denoted as $R_{clo}$, the angle between the orientation of $q_{0}$ and the tangent to the center $O_{clo}$ is denoted as $\beta_{\max}$:

(40) $$R_{clo}=\sqrt{x_{clo}^{2}+y_{clo}^{2}}\;\beta_{\max}=\arctan(\frac{x_{clo}}{y_{clo}}).\;$$

### 5.2. Spatial Increments Relative to Circular Arc Path

As can be seen in Figure 11, that a segment of the continues-curvature path produces increments $\Delta_{1}$ and $\Delta_{3}$, horizontally, and increments $\Delta_{2}$ and $\Delta_{4}$,vertically, with respect to the circular arc path. Draw a line parallel to the Y-axis through the center of the circle $O_{clo}$, intersecting the circle $C\langle O_{clo},R_{\min}\rangle$ at C. Point C can be regarded as the starting point of circular arc path, the distance between it with the X-axis and Y-axis are $\Delta_{1}$, $\Delta_{2}$, separately

(41) $$\Delta_{1}=\sqrt{\pi /\sigma_{\max}}FrC(\sqrt{\frac{k_{\max}^{2}}{\sigma_{\max}}})-R_{\min}\sin\theta_{clo},\;\Delta_{2}=\sqrt{\pi /\sigma_{\max}}FrS(\sqrt{\frac{k_{\max}^{2}}{\sigma_{\max}}})+R_{\min}\left(\cos\theta_{clo}-1\right).$$

The first segment is symmetrical about the line passing through $O_{clo}$ with a slope of $\tan(\theta_{contact}/2+\pi /2)$, $C^{\prime }$ is the point of symmetry of C about that line, and its distance from the contact point is $d=\sqrt{\Delta_{1}^{2}+\Delta_{2}^{2}}$, The angle formed by the line between point $C^{\prime }$ and the contact point and the tangent line is $\gamma =\arctan(\Delta_{2}/\Delta_{1})$. From the trigonometric relationship:

(42) $$\Delta_{3}=d\cdot \cos(\theta_{contact}-\gamma ),\;\Delta_{4}=d\cdot \sin(\theta_{contact}-\gamma ).\;$$

The whole continuous curvature path presents a centrosymmetric relation with respect to the contact point. Therefore, the total horizontal and vertical increments relative to the simple circle arc path are:

(43) $$\Delta_{L}=2(\Delta_{1}+\Delta_{3})=2\left(\sqrt{\pi /\sigma_{\max}}FrC(\sqrt{\frac{k_{\max}^{2}}{\sigma_{\max}}})-R_{\min}\sin\theta_{clo}+d\cdot \cos(\theta_{contact}-\gamma )\right),\;\Delta_{W}=2(\Delta_{2}+\Delta_{4})=2\left(\sqrt{\pi /\sigma_{\max}}FrS(\sqrt{\frac{k_{\max}^{2}}{\sigma_{\max}}})+R_{\min}\left(\cos\theta_{clo}-1\right)+d\cdot \sin(\theta_{contact}-\gamma )\right).$$

In addition, when the radius of the first arc is larger than the minimum turning radius, the two arcs should be considered separately, through the above analysis method can also obtained the corresponding conclusions easily.

**Proof** **of** **Theorem** **3.**  

Calculation of Minimum Internal and External Parking Space

**Figure 12:**
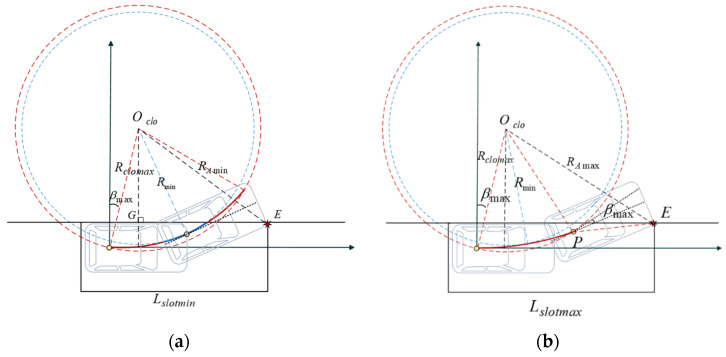
Minimum parking space in two cases: (**a**) lower bound; (**b**) upper bound.

The vehicle travels on the continuous-curvature path between two extreme circles (radius of $R_{\min}$ and $R_{clo}$), shown in Figure 11, so the minimum internal and external parking space both have upper and lower bounds. To make the required parking space as small as possible, it is assumed that at the end of the parking, the vehicle is parked close to the left boundary of the parking space ($W_{left}=0$). We will tackle the problem by taking into account the process of retrieving vehicles from the parking slot based on non-collision conditions. In the exit procedure, as shown in Figure 12, when the vehicle’s right front corner A moves to the line $\bar{O_{clo}E}$, if the vehicle’s reference point is located at the circular arc segment, the minimum parking slot reaches the lower bound in this situation, otherwise, if the reference point is located at the point of minimum curvature on the clothoid arc, the minimum parking slot reaches to the upper bound.

Based on the geometric analysis in the previous subsection and the application of the Pythagorean theorem to the triangle $O_{clo}GE$, the lower and upper bounds of the minimal length of the parking slot are:

(44) $$L_{slot}^{\min}=L_{bh}+\Delta_{1}+\sqrt{R_{A\min}^{2}-{\left(\Delta_{2}+R_{\min}-W_{v}/2\right)}^{2}}\;L_{slot}^{\max}=L_{bh}+\Delta_{1}+\sqrt{R_{A\max}^{2}-{\left(\Delta_{2}+R_{\min}-W_{v}/2\right)}^{2}}$$

where $R_{A\max}$ can be calculated in the triangle $O_{clo}PE$ by the cosine theorem

(45) $$R_{A\min}=\sqrt{{\left(R_{\min}+W_{v}/2\right)}^{2}+{\left(L_{v}-L_{bh}\right)}^{2}}\;R_{A\max}={\left(\sqrt{R_{i}^{2}+d_{PA}^{2}-2\cos(\pi /2+\theta_{f}+\beta_{i})R_{i}d_{PA}}\right)}_{\max}=\sqrt{R_{clo}^{2}+d_{PA}^{2}-2\cos(\pi /2+\theta_{f}+\beta_{\max})R_{clo}d_{PA}}.$$

**Figure 13:**
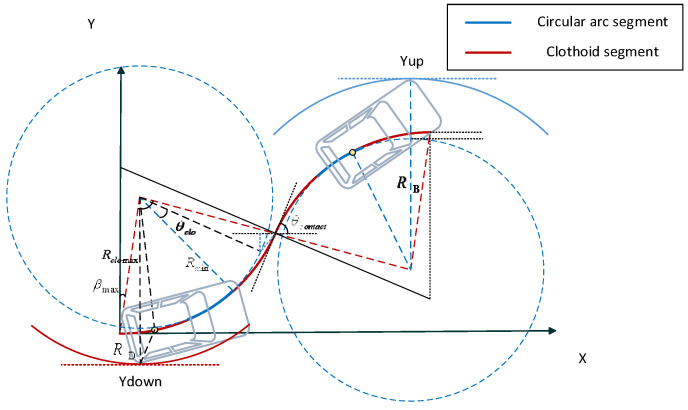
The trajectory of continuous curvature parking.

As shown in Figure 13, When the left front corner B of the vehicle moves to the parallel line of the Y-axis past the center of the circle $O_{clo}^{\prime }$, the reference point of the vehicle is located on the circular arc segment, the highest position $Y_{up}$ that the vehicle can reach during the parking maneuver is the smallest. Otherwise the reference point located on the clothoid arc segment, $Y_{up}$ is relatively larger. From the geometric relationship, it yields:

(46) $$Y_{up\min}=2R_{\min}(1-\cos\theta_{contact})+\Delta_{W}+R_{B\min}-(R_{\min}+\Delta_{2})\;Y_{up\max}=2R_{\min}(1-\cos\theta_{contact})+\Delta_{W}+R_{B\max}-(R_{\min}+\Delta_{2}),$$

(47) $$Y_{down\min}=-(R_{D\min}-(R_{\min}+\Delta_{2}))\;Y_{down\max}=-(R_{D\max}-(R_{\min}+\Delta_{2})).$$

So, the lower and upper bounds of the minimal width of the parking slot:

(48) $$W_{slot}^{\min}=W_{v}/2+R_{D\min}-(R_{\min}+\Delta_{2})\;W_{slot}^{\max}=W_{v}/2+R_{D\max}-(R_{\min}+\Delta_{2}).$$

The length of the minimum external parking space:

(49) $$L_{exter}=2R_{\min}\sin\theta_{contact}+\Delta_{L}+L_{v}-L_{bh}$$

The lower and upper bounds of the minimal width of the external parking space:

(50) $$W_{exter}^{\min}=2R_{\min}(1-\cos\theta_{contact})+\Delta_{W}+R_{B\min}-(R_{\min}+\Delta_{2})-W_{v}/2\;W_{exter}^{\max}=2R_{\min}(1-\cos\theta_{contact})+\Delta_{W}+R_{B\max}-(R_{\min}+\Delta_{2})-W_{v}/2.$$

where,

(51) $$R_{B\min}=\sqrt{{\left(R_{\min}+\frac{W}{2}\right)}^{2}+{\left(L-L_{bh}\right)}^{2}},\;R_{D\min}=\sqrt{{\left(R_{\min}+\frac{W}{2}\right)}^{2}+L_{bh}{}^{2}}\;R_{B\max}={\left(\sqrt{R_{i}^{2}+d_{PB}^{2}-2\cos(\pi /2+\theta_{f}+\beta_{i})R_{i}d_{PB}}\right)}_{\max}=\sqrt{R_{clo}^{2}+d_{PB}^{2}-2\cos(\pi /2+\theta_{f}+\beta_{\max})R_{clo}d_{PB}}\;R_{D\max}={\left(\sqrt{R_{i}^{2}+d_{PD}^{2}-2\cos(\pi /2+\theta_{b}+\beta_{i})R_{i}d_{PD}}\right)}_{\max}=\sqrt{R_{clo}^{2}+d_{PD}^{2}-2\cos(\pi /2+\theta_{b}+\beta_{\max})R_{clo}d_{PD}}\;\theta_{f}=\arctan\left(\frac{W/2}{L-L_{bh}}\right),\;\theta_{b}=\arctan\left(\frac{W/2}{L_{bh}}\right).$$

The proof of Theorem 3 is completed. □

### 5.3. Discussions

**Figure 14:**
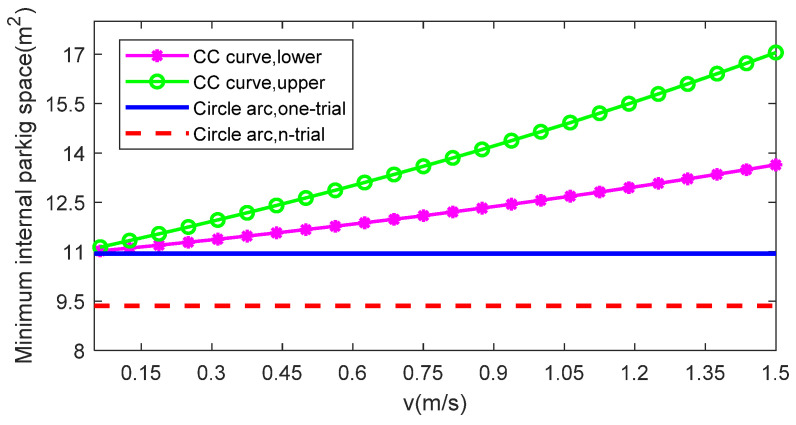

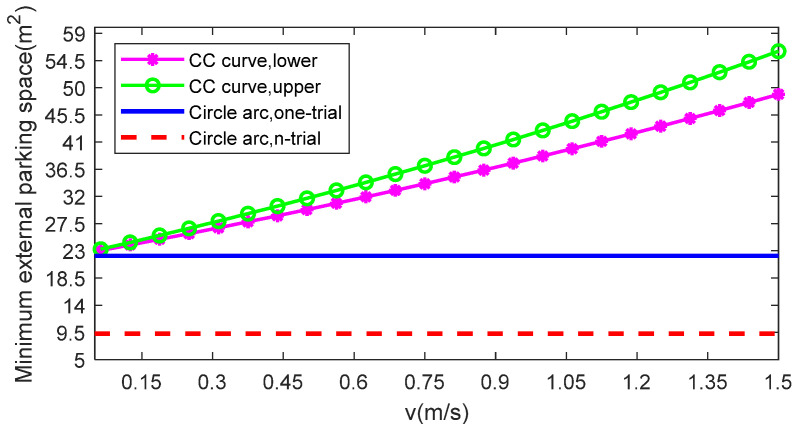
Comparison of the minimum internal and external parking space required by different parking methods.

**Figure 15:**
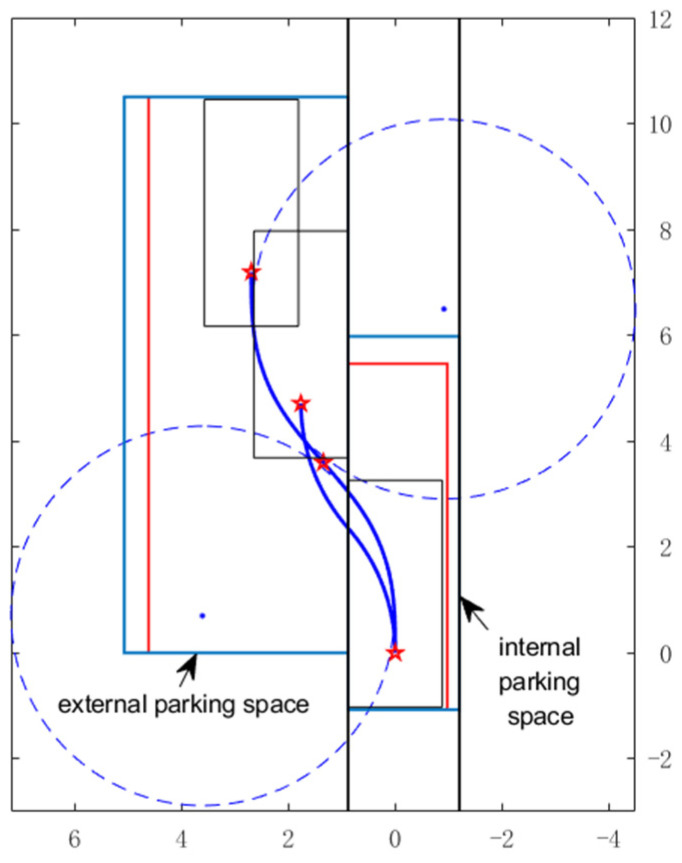
For the comparison between parking with continuous-curvature curve and circular arcs.

Figure 14 shows the upper and lower limits of the minimum internal and external parking space required for continuous-curvature (CC) parking in one trial as a function of vehicle velocity, and compares them to the minimum space required for one-trial and n-trial circular arc parking ($\Delta_{s}$ = 0.5 m). As can be seen, the space required for parking in one-trial is considerably bigger than parking in n-trial, so when the available space detected by the automatic parking system is smaller than the space required by the one-trial parking based on circular arc but larger than the n-trial parking, parking in several trials should be considered for path planning. However, when the detected space is big enough and the vehicle is parked at low speed, we prefer to plan a continuous-curvature path to parking, thus reducing the wear of the vehicle steering column and increasing driving comfort. Curvature continuity is a desirable property. The simulation on Matlab of the vehicle parking based continuous-curvature curve and circular arc is shown in Figure 15 when v is kept constant as 1 m/s., and the red and light blue rectangle area represents the lower limit and upper limit of space required by continuous curvature parking, respectively.

## 6. Conclusions

In this paper, the minimum parking space required for automatic parallel parking was considered based on a geometric analysis approach, which is an important prerequisite for future automatic parking systems. First, without limiting the number of parking maneuvers, the minimum internal and external parking spaces required for automatic parallel parking based on circular arc were given, and the conclusions were presented as closed-form solutions. Without loss of generality, the minimum parking spaces are only related to vehicle characteristics and mechanical properties, and have nothing to do with environmental factors. In addition, the minimum internal and external parking spaces are all approximately equivalent to the size of the bounding rectangle of the vehicle when vehicle performance constraints are discounted. Furthermore, for the classical one-trial parking based on simple admissible circular arcs and the improved parking based on continuous-curvature curve, the computational formulas of the minimum parking space were given, while the latter was presented as a set of upper and lower bounds in closed form. At last, the minimum internal and external parking space required for the different parking methods mentioned in this paper were compared and discussed. For the automatic parking system, a suitable path planning method can be selected by comparing the minimum parking space and the detected space to guide the vehicle to complete the parking maneuver.

The results of this paper are only for automatic parallel parking of small and medium-sized cars. In the future, the research will be extended to other vehicle configurations with more complex vehicle characteristics and kinematic models, such as articulated vehicles. Associated work is already on the agenda.

## Figures and Tables

**Table 1:** Parameters of used vehicle model.

Notation	$L_{v}$	$W_{v}$	$L_{w}$	$L_{fh}$	$L_{bh}$	$\delta_{max}$	$v_{\delta }$
Value	4235 (mm)	1765 (mm)	2510 (mm)	700 (mm)	1025 (mm)	35 (°)	25 (°/s)

**Table 2:** Minimum parking space under different $\Delta_{s}$.

$\Delta_{s}\;(m)$	0	0.100	0.250	0.500	0.750	1.000
n	Inf	2531	405	102	45	26
$L_{slot}^{\min}$, $L_{exter}^{\min}$	4.235	4.335	4.485	4.735	4.985	5.235
$W_{slot}^{\min}$	1.765	1.8094	1.8749	1.9759	2.0813	2.1560
$W_{exter}^{\min}$	1.765	1.8086	1.8698	1.9605	2.0369	2.0990

**Table 3:** Variation of external parking space dimension data with $W_{left}$.

$W_{left}\;(m)$	$\theta_{S_{min}}\;({}^{\circ})$	$W_{out,min}\;(m)$	$L_{out,min}\;(m)$	$Area_{out}\;(m^{2})$	$L_{slot}\;(m)$
0	41.081	2.799	7.922	22.181	5.817
0.1	42.284	2.800	7.306	20.457	5.872
0.2	43.430	2.799	7.121	19.931	5.924
0.3	44.633	2.801	7.010	19.637	5.974

## Data Availability

Not applicable.
